# Utility of the over-the-scope-clip system for treating a large esophageal perforation

**DOI:** 10.1007/s10388-014-0462-4

**Published:** 2014-09-28

**Authors:** Hiroyuki Ono, Masaki Tanaka, Kohei Takizawa, Naomi Kakushima, Noboru Kawata, Kenichiro Imai, Kinichi Hotta, Hiroyuki Matsubayashi

**Affiliations:** Division of Endoscopy, Shizuoka Cancer Center, Shimonagakubo 1007, Nagaizumi-cho, Sunto-Gun, Shizuoka 411-8777 Japan

**Keywords:** Clips, Esophagus, Endoscopic surgical procedure, Esophageal perforation

## Abstract

We report here a case of esophageal perforation made by an endoscope while treating cicatrical stenosis that developed after wide circumferential dissection of superficial esophageal carcinoma. Perforation closure with a conventional endoclip was difficult as the perforation was large and the surrounding tissue was fragile as a result of steroids administration for stenosis prevention. To avoid surgical intervention, we employed the over-the-scope-clip system and successfully closed the perforation. The favorable outcome suggests the utility of the over-the-scope-clip system for closing perforations when conventional methods are ineffective.

## Introduction

Endoscopic submucosal dissection (ESD) was developed in Japan for the treatment of early gastric cancer [1], and it is now also widely used to treat superficial esophageal cancer [2]. Since ESD enables the removal of large cancerous lesions from the superficial layer of the esophagus, the indications for endoscopic dissection in guidelines for the diagnosis and treatment of esophageal cancer have been revised: the size limitation for removable lesions (≤two-thirds of the circumference) by endoscopic techniques has now been lifted [3].

On the other hand, because esophageal stenosis is almost inevitable after the removal of subcircumferential and circumferential lesions, esophageal wall perforation during endoscopy and endoscopic balloon dilatation for the alleviation of stenosis is of particular concern in some cases [4].

Here we present a case where conventional endoclip closure of a large perforation of the stenotic esophagus was not possible and non-surgical closure was achieved using the over-the-scope-clip (OTSC) system (Ovesco Endoscopy, GmbH, Tuebingen, Germany).

## Case report

**Fig. 1:**
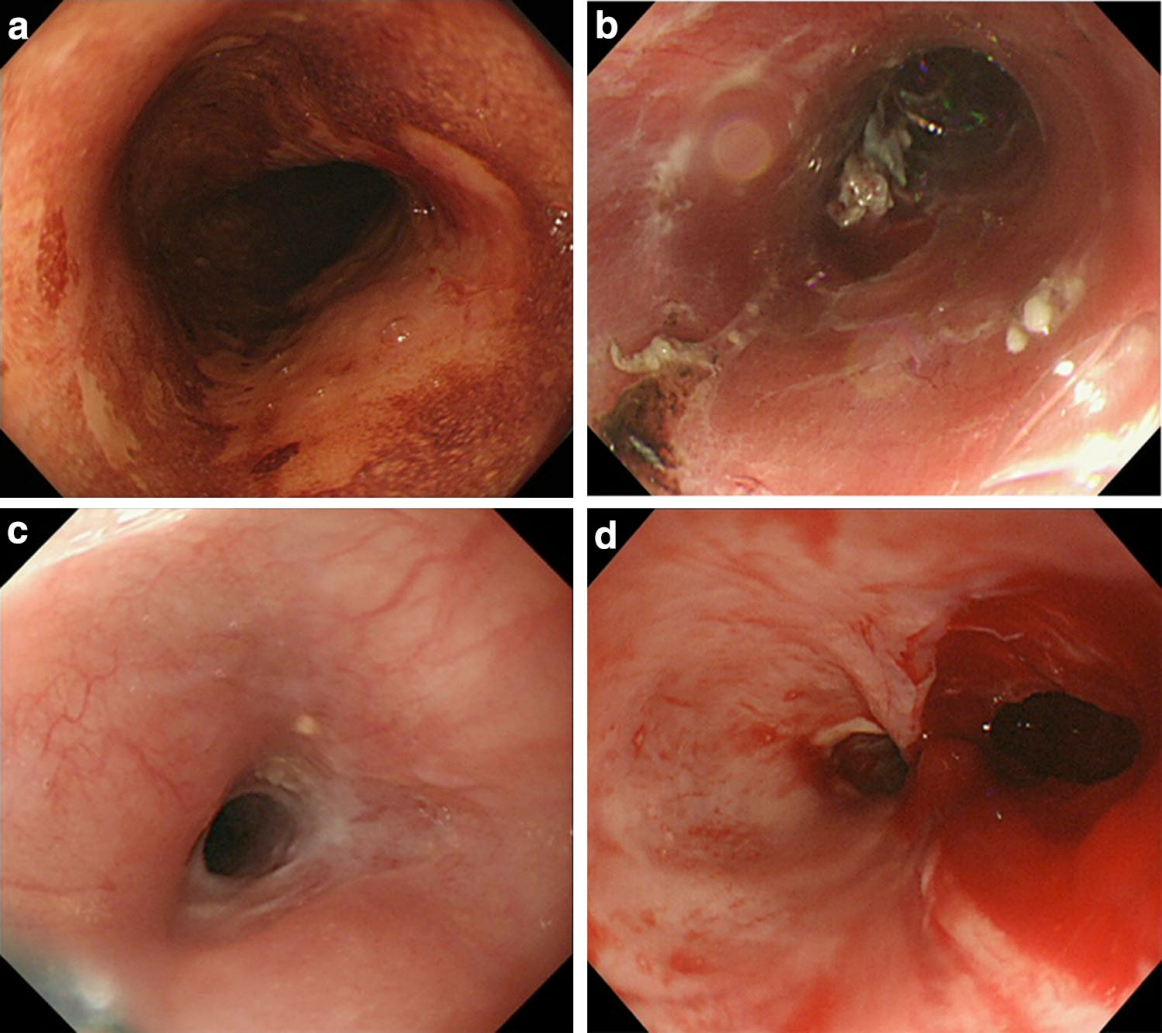
Endoscopic submucosal dissection (ESD) and an ulcer showing stenosis and perforation. **a** Superficial esophageal carcinoma about three-fourths the circumference of the esophagus located in the upper thoracic esophagus. **b** Immediately after esophageal endoscopic submucosal dissection (ESD), circumferential dissection was performed. **c** Cicatrical stenosis 3 months after esophageal ESD. **d** Large perforation due to the endoscope penetrating the wall in the upper-right side of the stenotic area

A 64-year-old woman presented with subcircumferential superficial esophageal carcinoma three-fourths the circumference of the esophagus in the upper thoracic esophagus (Fig. 1a). The depth of tumor invasion was evaluated as cT1a-MM by white light observation, narrow band imaging with magnifying endoscopy, and endoscopic ultrasound. No lymph node metastasis was evident on computed tomography (CT) images. Preoperative diagnosis was 0-IIc, cT1a-MM, 40 mm, cN0, cStage 0. According to the Japanese guidelines for the diagnosis and treatment of esophageal cancer, the Cancer Board for esophageal cancer of our hospital decided to perform ESD [3]. After obtaining informed consent, ESD was performed using an IT knife nano (KD-612, Olympus Medical Systems, Tokyo, Japan). Circumferential dissection (Fig. 1b) was necessary and to prevent the likely onset of stenosis, she received a local injection of triamcinolone acetonide (100 mg) into the submucosa underlying the ulcer immediately after ESD and was started on oral predonine therapy (30 mg/day) 1 week after ESD, which was tapered over a 2-month period. Histopathology findings indicated squamous cell carcinoma (0-IIc type, T1a-MM, ly1, v0, HM(−), and VM(−)). Additional treatment was considered because of the high risk for lymph node metastasis suggested by a positive finding of lymphatic involvement.

Two months after ESD, upper endoscopy revealed a residual ulcer, but no stenosis. However, stenosis was detected 1 month later (Fig. 1c). An endoscope (distal end external diameter, 9.8 mm) was inserted to traverse the stenosis but it penetrated the wall through to the mediastinum, leaving a perforation in the upper-right side of the stenotic area (Fig. 1d). Attempted closure with an endoclip (Olympus Medical Systems, Tokyo, Japan) was unsuccessful due to the large size of the perforation and fragility of the ulcer floor.

**Fig. 2:**
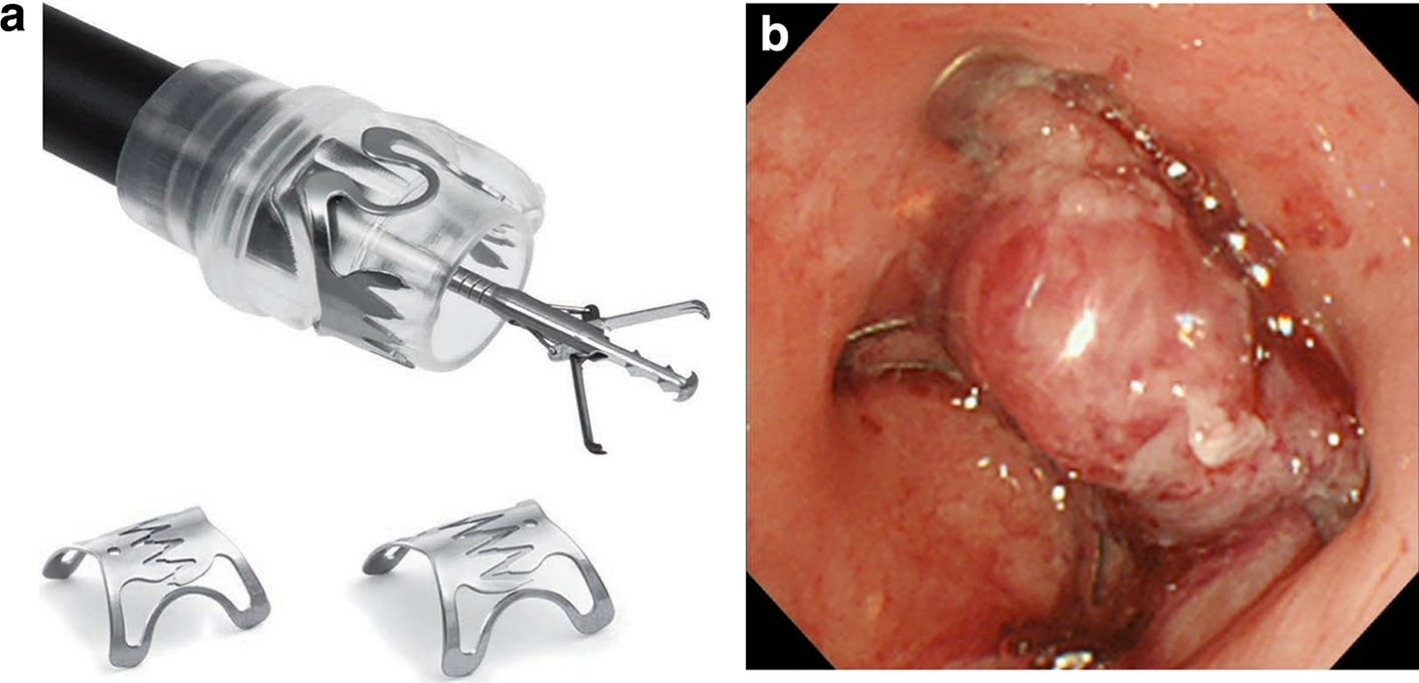
Suturing devices and perforation closure. **a** The over-the-scope-clip (OTSC) system showing the OTSC Twin Grasper with its branches open outside the endoscope and two clips (image reproduced with the permission of Century Medical Inc., Tokyo, Japan). **b** The perforation was closed by clipping the muscular layer with the OTSC system

**Fig. 3:**
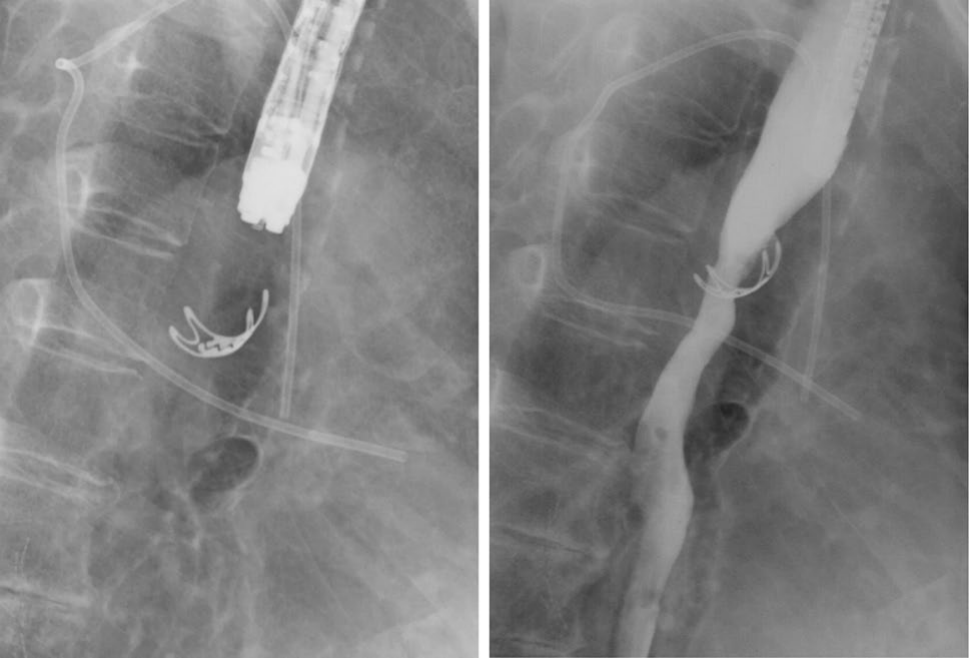
Non-contrast radiography showing the OTSC system in use (*left*) and contrast radiography showing no leakage (*right*)

**Fig. 4:**
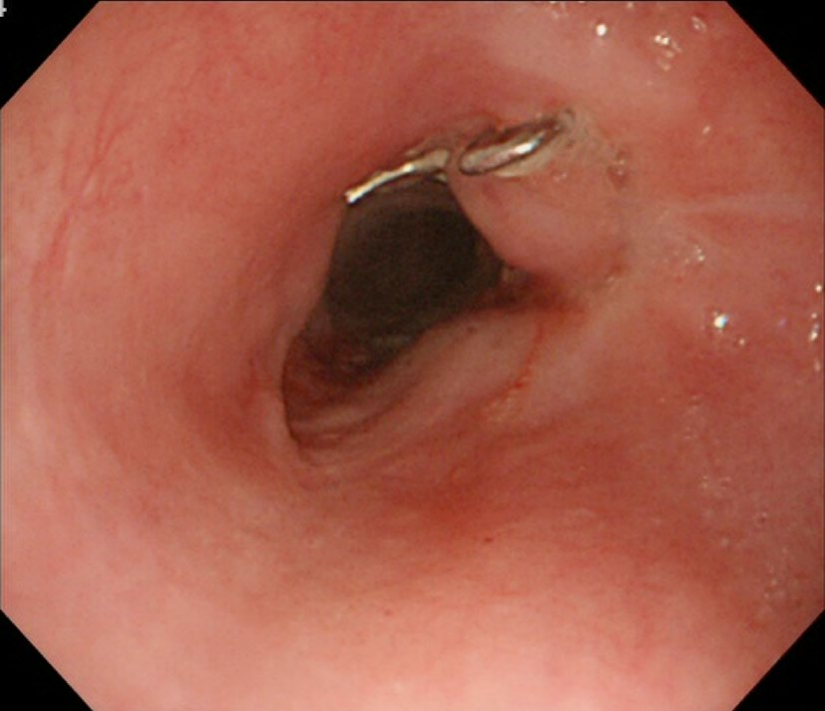
Endoscopic image taken 2 months after completing chemotherapy. The endoscope could traverse the site despite the presence of minor stenosis

We therefore employed the OTSC system to close the perforation (Fig. 2a). The branches of the OTSC Twin Grasper were released to grasp the sides of the lesion. Because the perforation hole had gaping edges, I opened one branch and grasped one edge of the perforation first. Then I opened the other branch and maneuvered the grasper to the other edge and grasped it, and then retracted into the attachment to place the clip (Fig. 2b). Closure was deemed successful. The patient fasted and received antibiotics, and her only complaint was mild chest discomfort. She did not develop fever, and both her leukocyte count and C reactive protein level were within normal range. Contrast radiography performed 5 days after the OTSC procedure revealed no leakage (Fig. 3). Oral intake of food and fluid was resumed and 3 weeks after contrast radiography her condition was sufficiently improved. After discussing the option of surgical treatment which is standard for her with our thoracic surgeon, the patient declined surgery and opted instead for CRT. Because re-examination by CT revealed no metastasis, preventive CRT was considered still effective, despite treatment was delayed by 1 month. Then she was completed CRT. At endoscopy 2 months after completing chemotherapy, the endoscope with a conventional-sized distal tip (9.8 mm) could traverse the site despite the presence of minor stenosis (Fig. 4). The patient reported no difficulties with nutritional intake. Because mild chest discomfort continued due to the OTSC, the clip was cut by argon plasma coagulation and removed. Thereafter, her complaints disappeared.

## Discussion

The number of cases of intraoperative perforation has increased as esophageal ESD has becomes more widespread, and perforation is frequently caused by endoscopic balloon dilatation used to alleviate stenosis, a complication of endoscopic dissection of large lesions [5]. Conservative therapy, such as perforation closure with an endoclip or placement of a nasogastric tube, is effective when the perforation is relatively small. Most perforations can typically be treated conservatively by fasting and administration of antibiotics. With a large perforation, serious mediastinitis and potentially fatal empyema may develop and closure is therefore crucial but often difficult.

The OTSC system is a new endoscopic tool used for non-surgical treatment of gastrointestinal perforation, fistula, and anastomotic leak [6]. It is the first effective system for closing a fistula endoscopically and it is therefore attracting interest [7]. To our knowledge, this is the first report of the OTSC system being used to treat a large perforation of a stenotic lesion that developed after esophageal ESD. Perforation of large lesions like this, which will likely have hard fibrotic edges and fragile surrounding tissue due to steroid treatment, occasionally need to be treated by surgical drainage or thoracotomy because of the difficulties in closing them with conventional endoclips. The present case demonstrates that the OTSC system can be used to close a large perforation of a stenotic site with good postoperative progress. Moreover, this non-surgical treatment maintains quality of life for patients.

There are several difficulties associated with using the OTSC system for perforation closure. Rotation of the OTSC Twin Grasper is not perfect and the target tissue sometimes cannot be grasped. Also, once clips are placed, they are difficult to remove. Special care is required when employing the OTSC system in organs with a narrow lumen, such as the esophagus and duodenum, because of the possible risk of stenosis. Despite these limitations, the OTSC system allows for the closure of large perforations not amenable to closure with conventional methods. As demonstrated in the present study, the OTSC system has good utility in difficult cases and thus warrants consideration.
